# Research on Pipeline Stress Detection Method Based on Double Magnetic Coupling Technology

**DOI:** 10.3390/s24196463

**Published:** 2024-10-07

**Authors:** Guoqing Wang, Qi Xia, Hong Yan, Shicheng Bei, Huakai Zhang, Hao Geng, Yuhan Zhao

**Affiliations:** 1 School of Information Science and Engineering, Shenyang University of Technology, Shenyang 110870, China; xiaqi158@smail.sut.edu.cn (Q.X.);; 2 Liaoning Nonferrous Geological Exploration Institute Limited Liability Company, Shenyang 110013, China

**Keywords:** oil and gas pipelines, strong and weak magnetic technology, stress detection, force-magnetic coupling, J-A model

## Abstract

Oil and gas pipelines are subject to soil corrosion and medium pressure factors, resulting in stress concentration and pipe rupture and explosion. Non-destructive testing technology can identify the stress concentration and defect corrosion area of the pipeline to ensure the safety of pipeline transportation. In view of the problem that the traditional pipeline inspection cannot identify the stress signal at the defect, this paper proposes a detection method using strong and weak magnetic coupling technology. Based on the traditional J-A (Jiles–Atherton) model, the pinning coefficient is optimized and the stress demagnetization factor is added to establish the defect of the ferromagnetic material. The force-magnetic relationship optimization model is used to calculate the best detection magnetic field strength. The force-magnetic coupling simulation of Q235 steel material is carried out by ANSYS 2019 R1 software based on the improved J-A force-magnetic model. The results show that the effect of the stress on the pipe on the magnetic induction increases first and then decreases with the increase in the excitation magnetic field strength, and the magnetic signal has the maximum proportion of the stress signal during the excitation process; the magnetic induction at the pipe defect increases linearly with the increase in the stress trend. Through the strong and weak magnetic scanning detection of cracked pipeline materials, the correctness of the theoretical analysis and the validity of the engineering application of the strong and weak magnetic detection method are verified.

## 1. Introduction

With the rapid development of national industrial construction, the demand for industrial energy has increased sharply. Oil and gas energy are usually transported to market users through pipeline transportation. Pipeline transportation is mostly underground, and the pipeline calibre is large to satisfy the requirements of low-cost consumption of energy transportation, stable working environment, and large transportation volume [1]. To advance the realization of an energy production and consumption revolution, oil and gas pipelines have become critical infrastructure for the transportation of petroleum and natural gas [2]. Energy transformation has become one of the reasons for the vigorous development of the national economy. However, the pipeline accident rate caused by this transformation has increased because of the associated increase in the total mileage and calibre, and the damage caused by pipeline accidents is considerable. Once a pipeline fails, it can result in a significant loss of life and property, as well as environmental pollution [3]. Oil and gas pipelines are in the soil environment for a long time, and the acid, alkali and minerals in the soil corrode the outer wall of pipelines and cause defects. The movement of the geological layer also causes pipeline displacement deformation and stress. When pipe wall corrosion occurs, the defective area of the pipe wall is stressed by the pressure of the carrying medium. Under the continuous action of stress on the defects, the fatigue strength, brittle fracture resistance and high-temperature creep cracking ability of pipeline materials are reduced, and their material properties weaken, resulting in pipeline leakage, explosion and other accidents. These accidents result in substantial production losses and diminished economic security. Therefore, pipe stress concentration and pipe wall corrosion defects directly affect the safe operation of pipelines [4]. Regular pipeline repair and maintenance are required to ensure pipeline safety. According to pipeline data, the incidence of pipeline accidents increases exponentially with the increase in the pipeline undetected interval. The detection of defective stress in pipelines is an essential production process requirement because it can ensure the normal operation of oil and gas transportation [5].

Safety assessments of the corrosion state and material stress of the inner and outer walls of pipelines have been realised through the research, development and application of in-pipeline detection technology. Active maintenance of the risk area is predicted in advance to prevent pipeline accidents, and professional maintenance plans are designed based on the degree of metal damage to achieve scientific and reliable pre-inspection of pipeline safety risks [5,6,7]. Pipeline risks mainly exist during pipeline construction and operation, so regular in-pipeline inspection from the laying of pipelines is recognised by the pipeline industry as an effective means to ensure the safety of pipeline structures [8,9].

At present, the actual application of non-destructive testing technology includes ultrasonic testing, X-ray inspection, magnetic leakage detection, weak magnetic detection, and eddy current testing technology. These technical means can effectively evaluate materials for metal-material structural equipment, such as oil and gas pipelines and pressure storage vessels. They can be used for quality testing and to analyse safety risks for accident prevention [10]. The main purpose of traditional non-destructive testing technology is to detect the volume defects and cracks of equipment materials. However, its stress detection and recognition abilities are low, the welded pipe body cannot be effectively detected, the pressure of the medium in the pipe and the stress concentration area caused by external factors of the soil cannot be assessed, and the stress safety factors cannot be specifically evaluated, easily leading to pipeline failure caused by metal stress fatigue damage [11,12]. Through weak field detection technology, the stress state at the pipeline defect can be detected. Weak magnetic detection technology evaluates the magnitude of stress through the magnetic change in the inspected area; the stress acting on the material area leads to a change in metal permeability, and the stress concentration area can then be evaluated and detected early in accordance with the signal characteristics [4,13]. Traditional weak magnetic detection technology does not require magnetisation equipment to provide an excitation magnetic field, as it instead uses the earth’s magnetic field as the detection environment. The magnetic signal generated is too weak and is easily disturbed by external magnetic fields or signals, so the detection results have numerical errors and wrong evaluations [14]. Defects in pipe volume also change the field-weakening detection signal, and defects and stress signals cannot be identified during inspection [15,16]. The mechanism of stress detection technology for magnetic defects involves applying a non-excited saturation magnetic field to the ferromagnetic material under investigation. This approach enhances the detection signal, ensuring that stress signals are not obscured under unsaturated magnetic fields. Consequently, it enables the detection of combined signals of pipeline defects and stress.

In this study, defect stress detection of ferromagnetic pipes is investigated using the strong and weak magnetic coupling detection method, and the mechanism of strong and weak magnetic detection and the magnetic strength signal characteristics of the defect stress of ferromagnetic materials are examined by combining metal-materials science and ferromagnetism. The coefficient and demagnetisation coefficient of the Jiles–Atherton (J-A) force–magnetic model are optimised, the force magnetic relationship model is solved, and optimal detection of magnetic field strength is proposed. Through the finite element simulation of force–magnetic coupling, the relationship between the magnetic induction intensity and stress of pipeline materials under different excitation intensities is obtained, and the law of the influence of stress on magnetic induction intensity is determined. A force–magnetic correlation experiment is conducted on a Q235 pipe test piece to verify the correctness of the theoretical model.

## 2. Force–Magnetic Coupling Model Based on Strong and Weak Magnetic Technology

### 2.1. Improvement of the J-A Force–Magnetic Model

The key to successful research on pipeline defect stress detection technology using the strong and weak magnetic coupling method is the theory of this method, which is based on magnetomechanics model theory. The J-A hysteresis model describes the relationship between magnetisation and magnetic field strength [17]. Two differential equations describing irreversible differential magnetic susceptibility and reversible differential magnetic susceptibility are derived by studying the motion mechanism of the domain wall, and the initial magnetisation curve and hysteresis loop theoretical model under a stress field are obtained using a suitable hysteresis-free magnetisation function.

Magnetisation intensity *M* is converted into an actual detected magnetic signal, that is, magnetic induction intensity *B*, which is converted based on the relationship between magnetic induction intensity and magnetisation strength [18].

(1) $$B=\mu_{0}\left(H+M\right)$$

where $\mu_{0}$ is vacuum permeability, M denotes magnetisation strength, and H refers to magnetic field strength.

The Langevin equation is suitable for simulating the hysteresis-free state of ferromagnetic materials because of its inherent accordance with magnetic quantum theory:

(2) $$L_{\left(x\right)}=cothx-\frac{1}{x}$$

When *x* approaches 0, *L*_(*x*)_ = 0; when *x* approaches infinity, *L*_(*x*)_ = 1. The J-A model uses the Langevin equation to simulate the hysteresis-free curve of the average field under ideal conditions, and the hysteresis-free magnetisation intensity, *M_an_*, is expressed as:

(3) $$M_{an}=M_{s}\left(coth\left(\frac{H_{e}}{a}\right)-\frac{a}{H_{e}}\right)$$

where *M_s_* is the saturation magnetisation strength of the material, *a* is the shape parameter of the hysteresis-free magnetisation curve given the dimension of the magnetic field, and *H_e_* is the equivalent magnetic field of the combination of the applied magnetic field and the magnetic field generated by the material itself. The last variable can be expressed as:

(4) $$H_{e}=H+\alpha M$$

where *H* is the external magnetic field strength, *M* is the magnetisation strength, and α is the average field coupling parameter that reflects the coupling relationship between magnetic domains.

Magnetisation intensity *M* is expressed by reversible magnetisation and irreversible magnetisation as follows:

(5) $$M=M_{irr}+M_{rev}$$

where $M_{irr}$ is an irreversible component caused by magnetic domain containment formed by the discontinuity of the material structure, and $M_{rev}$ is a reversible component caused by the elastic bending of the magnetic domain [19]. The irreversible magnetisation process of ferromagnetic materials is responsible for hysteresis and the formation of hysteresis loops.

The pinning of the magnetic domain wall by the pinning point leads to irreversible magnetisation, and the energy ($E_{pin}$) consumed by the pinning process can be expressed as:

(6) $$E_{pin}=\mu_{0}k\int_{0}^{M_{irr}}d_{M_{irr}}$$

where *k* is the material pinning coefficient, and its representative dimension is the A/m hysteresis characteristic coefficient. The pinning coefficient directly acts on the coercive force field and is closely related to the density of the pinning point and wall displacement.

From an energy point of view, the presence of stress increases stress, domain wall, and demagnetisation energies [20]. For positive magnetostrictive materials, when tensile stress is applied, the magnetization direction shifts to the tensile stress direction, that is, the stress axis becomes the easy-magnetisation axis, thereby enhancing the magnetisation of the tensile stress direction. Assuming that the original ferromagnetic specimen is composed of four magnetic domains, when the tensile stress is small, the volume of the magnetic domain perpendicular to the tensile stress direction decreases; as the stress increases, this part of the magnetic domain is eventually eliminated entirely, and the magnetoelastic energy is minimised. The effect of compressive stress is exactly the opposite; at this time, the stress axis becomes the difficult-magnetisation axis, that is, the magnetisation direction shifts to the direction perpendicular to the compressive stress, thereby enhancing the magnetisation perpendicular to the compressive stress direction. Mechanical stress affects the coercive force field and loss density [21]. When the magnetic field strength increases, the domain volume decreases, and the domain wall displacement increases. Given nonhysteretic magnetisation and the corresponding magnetic domain volume fraction distribution, the relationship between the optimised pinning coefficient *k* and hysteresis-free magnetisation strength can be expressed as:

(7) $$k=k_{0}\left(1-k_{r}\frac{M_{an}}{M_{s}}\right)$$

where $k_{0}$ is the initial pinning coefficient and $k_{r}$ is the relative coefficient.

According to the conservation of energy theory, the actual magnetisation energy is equal to hysteresis-free magnetisation energy minus hysteresis loss energy, that is,

(8) $$M=M_{an}-\frac{k\delta }{\mu_{0}}\frac{d_{M_{irr}}}{d_{H_{e}}}$$

where δ is the direction coefficient. When $\frac{d_{t}}{d_{H}}>0$, δ = 1; when $\frac{d_{t}}{d_{H}}<0$, δ = −1. $M_{an}$ is the hysteresis-free magnetisation strength, where $\frac{d_{M_{irr}}}{d_{H_{e}}}$ is the rate of change of irreversible components with respect to the equivalent magnetic field. The relationship between reversible elastic component $M_{rev}$ and irreversible component $M_{irr}$ is

(9) $$M_{rev}=c\left(M_{an}-M_{irr}\right)$$

where *c* is the reversible component coefficient.

Equations (3), (4), (8) and (9) are used to derive the applied magnetic field. The relationship between magnetisation strength and the applied magnetic field is

(10) $$\frac{d_{M}}{d_{H}}=\frac{-\frac{\left(M_{an}-M\right)}{k\delta }-\frac{c}{1-c}\frac{d_{M_{an}}}{d_{H}}}{\frac{\alpha \left(M_{an}-M\right)}{k\delta }-\frac{1}{1-c}}$$

The relationship between the hysteresis-free magnetisation strength $M_{an}$ and the applied magnetic field *H* can be expressed as

(11) $$\frac{d_{M_{an}}}{d_{H}}=\frac{M_{s}\left[-csch^{2}\left(\frac{H_{e}\left(M_{an}\right)}{a}\right)\frac{1}{a}+\frac{a}{H_{e}^{2}\left(M_{an}\right)}\right]}{1+M_{s}\left[{csch}^{2}\left(\frac{H_{e}\left(M_{an}\right)}{a}\right)\frac{1}{a}-\frac{a}{H_{e}^{2}\left(M_{an}\right)}\right]\alpha }$$

Equations (10) and (11) are for the magnetisation strength of ferromagnetic materials under the action of external magnetic fields. As shown by the formula above, the external excitation magnetic field can change the magnetism of ferromagnetic materials and strengthen the magnetic signals generated by ferromagnetic materials.

Ferromagnetic materials change the internal effective field under stress, and this change has two parts; one part is magnetostrictive, and the other part affects the stress demagnetisation factor. When a material is subjected to mechanical stress, most magnetic domains rotate in a direction parallel to the stress, so a stress demagnetisation factor ($D_{\sigma }$) needs to be introduced. The relation of the stress demagnetisation factor with stress can be expressed as

(12) $$D_{\sigma }=\frac{3\lambda_{S}\sigma }{M_{S}B_{S}}$$

where $M_{s}$ is the saturation magnetisation intensity, and $B_{s}$ is the saturation magnetic induction intensity. The saturation magnetostriction coefficient $\lambda_{s}$ is expressed as

(13) $$\lambda_{S}\left(\sigma \right)=-\frac{2b\left(\sigma \right)\left(1+v\right)}{3E}$$

where υ is the Poisson’s ratio, E is the modulus of elasticity, and b(σ) is a stress-dependent function and can be expressed as

(14) $$b\left(\sigma \right)=\left\{\begin{array}{l} -be^{-\frac{1}{2}\left(\frac{\sigma }{\sigma_{0}}\right)},\sigma >0\\ b,\sigma <0 \end{array}\right.$$

where b and $\sigma_{0}$ are constants.

Let the whole system be subjected to external magnetic field H and unidirectional stress σ to obtain a corresponding equivalent magnetic field. The relationship between magnetisation strength M and the equivalent magnetic field $H_{e}$ can be expressed as

(15) $$H_{e}=H+\alpha M+\frac{3\sigma d_{\lambda }}{2\mu_{0}d_{M}}-D_{\sigma }M$$

where α is the magnetic domain coupling coefficient, $\mu_{0}$ refers to vacuum permeability, and the magnetostriction coefficient λ is given as

(16) $$\lambda =\sum_{i=0}^{i=\infty }\gamma_{i}M^{2i}$$

where γ is an expansion parameter. At the same time, the equivalent field $H_{e}$ is differentiated from *H* as

(17) $$\frac{d_{H_{e}\left(M\right)}}{d_{H}}=1+\left(\alpha +\frac{3\sigma {d^{2}}_{\lambda }}{2\mu_{0}d_{M^{2}}}\right)\frac{d_{M}}{d_{H}}-\frac{D_{\sigma }d_{M}}{d_{H}}$$

When the value of *i* is two, the magnetostriction coefficient λ is expressed as

(18) $$\lambda \approx \gamma_{1\left(\sigma \right)}M^{2}+\gamma_{2\left(\sigma \right)}M^{4}$$

Then, the differential equation of the equivalent field to the excitation intensity can be expressed as

(19) $$\frac{d_{H_{e}\left(M\right)}}{d_{H}}=1+\left\{\alpha +\frac{3\sigma }{2\mu_{0}}\left[2\gamma_{1\left(\sigma \right)}+12\gamma_{1\left(\sigma \right)}M^{2}\right]-D_{\sigma }\right\}\frac{d_{M}}{d_{H}}$$

where $\gamma_{i}^{n}\left(0\right)$ is the nth derivative of stress at *σ* = 0. After the experimental magnetostrictive measurement data are fitted, the coefficient stress-determining parameters $\gamma_{1}$ and $\gamma_{2}$ of the Taylor series can be numerically approximated and obtained by expanding the Taylor series as follows:

(20) $$\gamma_{i}\left(\sigma \right)=\gamma_{i}\left(0\right)+\sum_{n=1}^{\infty }\frac{\sigma^{n}}{n!}\gamma_{i}^{n}\left(0\right)$$

We take *n* = 1 and substitute Equation (20) into Equation (19) to derive

(21) $$\begin{array}{l} \frac{d_{H_{e}\left(M\right)}}{d_{H}}=\\ 1+\left\{\alpha +\frac{3\sigma }{\mu_{0}}\left[\left(\gamma_{1\left(0\right)}+{\gamma_{1}^{1}}_{\left(0\right)}\sigma \right)+6\left(\gamma_{2\left(0\right)}+{\gamma_{2}^{1}}_{\left(0\right)}\sigma \right)M^{2}\right]-D_{\sigma }\right\}\frac{d_{M}}{d_{H}} \end{array}$$

From $\frac{d_{M_{irr}}}{d_{H}}=\frac{d_{M_{irr}}}{d_{H_{e}}}\frac{d_{H_{e}}}{d_{H}}$ and Equations (3) and (21), we obtain

(22) $$\begin{array}{l} \frac{M_{an}-M}{k\delta }\left\{1+\left\{\alpha +\frac{3\sigma }{\mu_{0}}\left[\left(\gamma_{1\left(0\right)}+{\gamma_{1}^{1}}_{\left(0\right)}\sigma \right)+6\left(\gamma_{2\left(0\right)}+{\gamma_{2}^{1}}_{\left(0\right)},\sigma \right)M^{2}\right]-D_{\sigma }\right\}\right\}\frac{d_{M}}{d_{H}}\\ =\frac{1}{1-c}\left(\frac{d_{M}}{d_{H}}-c\frac{d_{M_{an}}}{d_{H}}\right) \end{array}$$

From the relationship between magnetisation intensity M and equivalent magnetic field $H_{e}$ in Equations (1) and (15), the differential formula of the magnetic induction intensity applied to the magnetic field can be derived as

(23) $$\frac{d_{M}}{d_{H}}=\frac{-\left(1-c\right)\mu_{0}\frac{M_{an}-\frac{M}{\mu_{0}}}{k\delta }-c\frac{d_{M_{an}}}{d_{H}}}{1-c\cdot \frac{M_{an}-\frac{M}{\mu_{0}}}{k\delta }\left\{\begin{array}{l} \alpha +\frac{3\sigma }{\mu_{0}}\left[\begin{array}{l} \left(\gamma_{1\left(0\right)}+{\gamma_{1}^{1}}_{\left(0\right)}\sigma \right)+\\ 6\left(\gamma_{2\left(0\right)}+{\gamma_{2}^{1}}_{\left(0\right)}\sigma \right)\\ {\left(\frac{B-\mu_{0}H}{\mu_{0}}\right)}^{2} \end{array}\right]\\ -D_{\sigma } \end{array}\right\}-1}$$

Equation (23) can be used to analyse the strong and weak magnetic signal characteristics of materials under stress by examining the numerical relationship between *M* and *H*, where $\frac{d_{M_{an}}}{d_{H}}$ is the rate of change in hysteresis-free magnetisation strength with respect to the applied magnetic field.

The initial magnetisation curve and the change in the hysteresis loop under different magnetisation stages are investigated to validate the force–magnetic model, and the function law is presented through images. MATLAB R2018b software is used to analyse the theoretical model, and the magnetisation curve is drawn by calculating Equation (23). The magnetic field interval is set to [0 A/m–20 KA/m], and the function curve of magnetic induction intensity with the change in magnetic field strength is calculated and plotted to study the change law of force–magnetic characteristics.

Q235 steel is adopted as an example. The selected parameters are as follows: saturation magnetisation strength $M_{S}=1.585\times 10^{6}\;A\cdot M^{-1}$; average field coupling parameter α=0.001; reversible component coefficient c=0.2; initial pinning coefficient $k_{0}=70$; expansion parameters $\gamma_{1}=7\times 10^{-18}\;A^{-2}{\cdot M}^{2}$, $\gamma_{1}^{\prime }=-1\times 10^{-25}\;A^{-2}{\cdot M}^{2}{\cdot \mathrm{Pa}}^{-1}$, $\gamma_{2}=-3.3\times 10^{-30}\;A^{-4}{\cdot M}^{4}$, and $\gamma_{2}^{\prime }=2.1\times 10^{-38}\;A^{-4}\cdot M^{4}\cdot {\mathrm{Pa}}^{-1}$; elastic modulus E=207 GPa; Poisson’s ratio ν=0.3 and hysteresis-free magnetisation curve shape parameter $a=1400\;{A\cdot M}^{-1}$.

**Figure 1:**
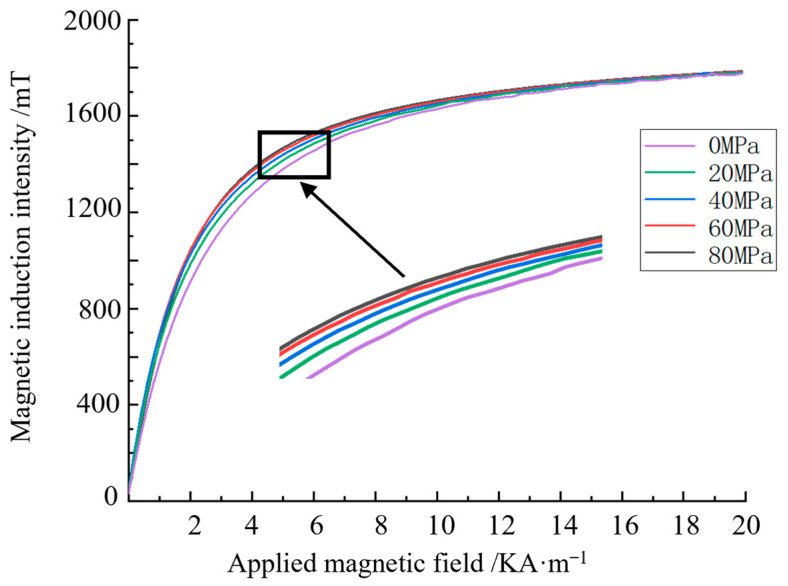
Magnetisation curves under different stresses.

The relationship curve between the applied magnetic field and the magnetic induction intensity under applied stress values of 0, 20, 40, 60, and 80 MPa is calculated and shown in Figure 1, where the abscissa is the applied magnetic field strength and the ordinate is the magnetic induction intensity.

When stress is applied to ferromagnetic materials, the stress causes the curvature of the magnetisation curve to increase, making the magnetisation curve shift upward. Under different applied magnetic field strengths, the stress action causes the magnetisation curve to shift upward at different degrees. The degree of the upward movement represents the influence of stress on the magnetic signal. With the increase in the applied magnetic field, the degree of the influence of stress on the magnetisation curve gradually increases then decreases. When the excitation is saturated, the stress has little effect on the magnetisation curve with and without coinciding stress.

### 2.2. Calculation of the Optimal Excitation Intensity for Strong and Weak Magnetic Stress Detection

**Figure 2:**
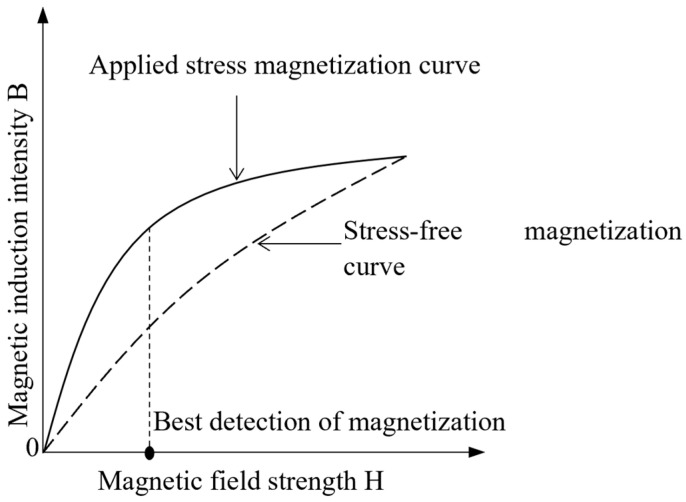
Schematic of the optimal detection of magnetisation.

Under a certain applied magnetic field strength, the difference in magnetic induction intensity between stress and no-stress conditions is used to indicate the signal generated by stress. The applied magnetic field strength corresponding to the maximum stress generation signal is called the optimal detection magnetic field strength. The detected stress generation signal is evident when the applied magnetic field results in an ideal detection of magnetic field strength. A schematic of the optimal detection of magnetisation is shown in Figure 2.

**Figure 3:**
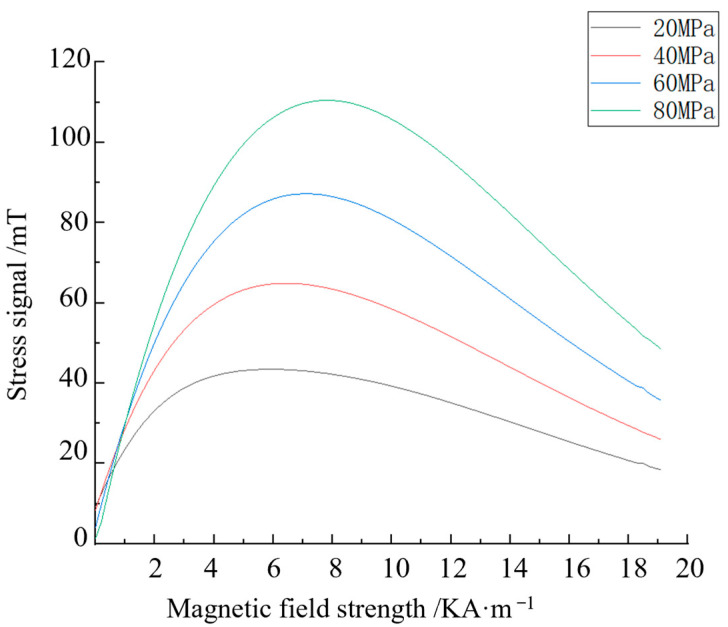
Change curve of the stress generation signal and applied magnetic field.

The magnetisation curves when 0, 20, 40, 60 and 80 MPa of stress are applied to Q235 steel are calculated using MATLAB R2018b software to solve the strong and weak magnetic detection model. The relationship between the stress generation signal and the applied magnetic field when different stresses are applied is calculated and shown in Figure 3, where the abscissa is the applied magnetic field strength, and the ordinate is the stress signal.

As indicated in Figure 3, when the applied magnetic field is 0 KA/m, the magnetic induction intensity corresponding to the different stress signals is 0 mT. Under a load of 80 MPa, When the applied magnetic field strength is in the range of 0–8 KA/m, the stress signal increases with the increase in excitation strength. When the applied magnetic field strength exceeds 8 KA/m, the stress signal decreases with the increase in excitation strength. When the applied magnetic field is 20 KA/m, the stress signal is close to 0. At the excitation stage, when the applied magnetic field strength is 8 KA/m, the stress signal has the maximum value, indicating that the stress signal recognition is high in this magnetic field environment, the stress is easy to detect, and the signal generated by stress in the magnetic saturation environment is small. The recognition of the stress signal is low, and the stress cannot be detected by the magnetic method. The stress change causes the maximum signal generated by stress to change slightly with the applied magnetic field strength.

**Figure 4:**
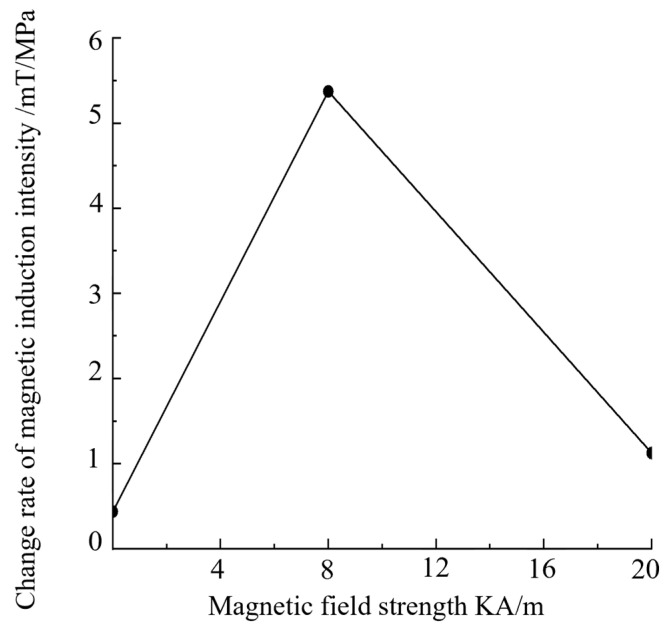
Relationship curve between magnetic field intensity and magnetic induction intensity change rate.

The correspondence between stress and magnetic induction intensity is studied in a non-excitation environment. The optimal detection excitation environment is 8 KA/m, and the value of the strong magnetic excitation environment is 20 KA/m. The relationship curve between stress and magnetic induction intensity is calculated with the strong and weak magnetic stress detection model, and the stress is set from 0 MPa to 80 MPa; the value is taken every 20 MPa, and the result is shown in Figure 4.

Figure 4 indicates that the magnetic signal increases with the increase in stress in the 8 KA/m magnetic field environment, and the increase trend is close to being linear within a certain stress range. The slope of the stress and magnetic signal fitting curve in the environments without a magnetic field and with a 20 KA/m strong magnetic field is almost zero, indicating that the stress in the strong magnetic field environment has almost no effect on the magnetic signal, whereas the stress under the weak magnetic field has a considerable effect on the magnetic signal.

## 3. Simulation Calculation of Strong and Weak Magnetic Stress Detection

A force–magnetic coupling finite element simulation model is established with ANSYS 2019 R1 simulation software, and the correspondence between the stress applied to the defective pipe and the stress at the defect under the action of external force is analysed. The sequential coupling method is used for calculation and analysis, and the changes in the magnetophysical properties of ferromagnets caused by the static simulation results are brought into the static magnetic field simulation to study the influence of the external magnetic field and stress on the stress signal characteristics at the defect for calculation and analysis.

**Figure 5:**
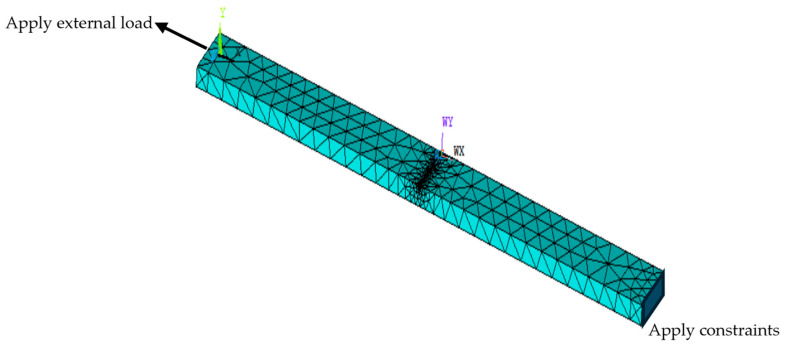
Defect steel mesh division model.

A 3D finite element model of Q235 steel is established. The model size is 600 mm in length × 60 mm in width × 15 mm in thickness, and the size of the intermediate penetration defect is 60 mm in length × 1 mm in width × 2 mm in depth. The model parameters are set as follows: according to the Chinese National Standard GB/T700-2006 [22], the elastic modulus of the material is set to 207 GPa, the yield strength is set to 235 MPa, and the Poisson’s ratio is set to 0.3. After the parameters are set, the geometric model is established by Boolean operations on the defect and pipe surface, as shown in Figure 5.

### 3.1. Static Simulation Analysis

A structure is selected to perform a static nonlinear simulation of the geometric model. The simulation module is the No. 96 ferromagnetic material analysis unit, which can accurately reflect the characteristics of mechanical changes to reveal the stress distribution of defects under load. After the model is defined, the left surface of the model is used as the fixed surface, and the right surface is employed as the applied tensile surface to simulate the force situation of the steel in the state of unidirectional stress. Tensile force perpendicular to the plane direction is applied to the applied surface. The meshing mode is set to be freely divided, and intelligent minimum meshing is used to ensure the accuracy of the simulation results, as shown in Figure 5.

**Figure 6:**
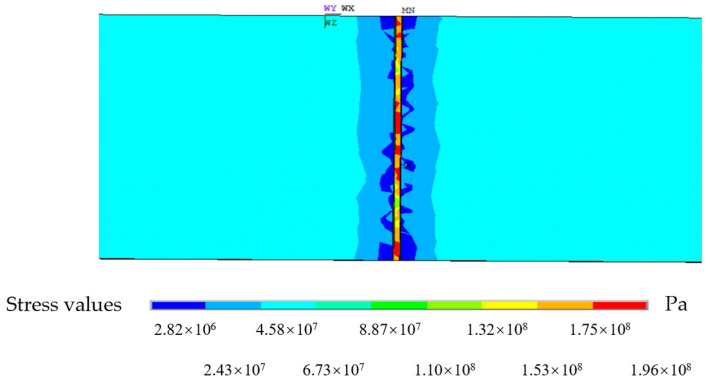
Stress cloud diagram of steel defects.

The load in the direction of the steel plate is applied on the left side of the model, the constraint is applied on the right side, the static cloud diagram is recorded when the external load is applied, and the accuracy of the simulation is guaranteed by multiple calculations. The defect part is enlarged, as shown in Figure 6.

As indicated in Figure 6, when the external load is applied to the steel plate, the stress is almost entirely concentrated near the defect and distributed symmetrically and radially.

### 3.2. Force–Magnetic Coupling Simulation Analysis

On the basis of the static simulation results, the numerical model of stress concentration change is obtained, and the No. 96 ferromagnetic material analysis unit is used to define the properties of air and steel. The air properties are given by permeability, and the permeability value is 1. The properties of the stressed specimen are defined by the B-H curve of the pipe under different stresses.

**Figure 7:**
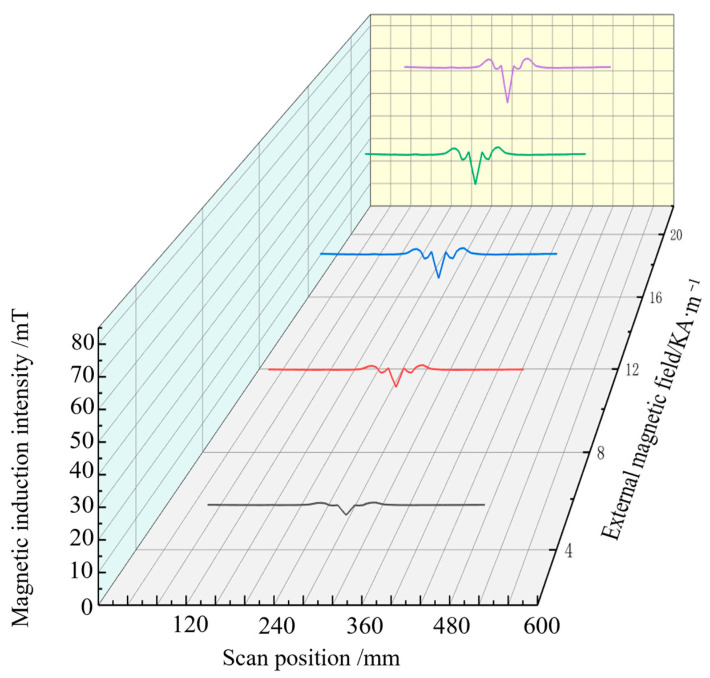
Axial composite signal.

**Figure 8:**
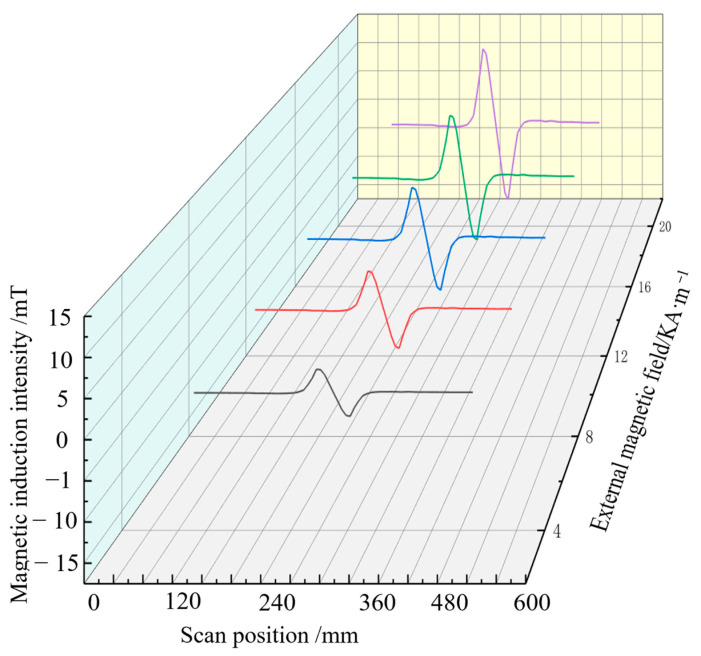
Radial composite signal.

The magnetic induction intensity signal along the length of the steel bar is extracted when the pipe model is subjected to 60 MPa stress and magnetised by different external magnetic fields. The axial component of the magnetic signal extracted in different magnetic field environments is shown in Figure 7, and the radial component change is given in Figure 8, where the abscissa is the magnetic signal scanning position, and the ordinate is the defect and stress composite magnetic component signal.

Figure 7 indicates that when tensile stress of 60 MPa is stably applied to the pipe model, and the axial component signal at the defect has the minimum value, the overall magnetic signal moves up with the increase in the external magnetic field strength, and the characteristic peak value becomes increasingly obvious.Changes in the axial and radial components of the magnetic signals of the pipe extracted in different magnetic field environments are shown in Figure 8. Figure 8 shows that the radial component signal at the defect has maximum and minimum values, and the signal crosses the zero point. As the strength of the external magnetic field increases, the magnetic signal moves up as a whole, and the characteristic peak-to-peak value increases. These results are consistent with ferromagnetic theory, and the magnetic induction intensity of ferromagnetic materials in the magnetic field changes, which verifies the ferromagnetic material strength and weak magnetic detection theory.

### 3.3. Calculation of the Optimal Detection Field Strength of Strong and Weak Magnetism

Strong and weak magnetic detection technology needs to select the magnetic signal at the unsaturated magnetisation stage, which includes the coupling of defect and stress influencing factors with a simple defect signal under a strong magnetic field. If the magnetic field strength selected at the coupling point is too low, the detection signal will be too weak and easily disturbed by external factors, the error will increase, and the stress effect will have little effect. If the magnetic field strength selected at the coupling point is close to magnetised saturation, the stress signal will be ignored, and only the defect signal will be measured. Therefore, determining the magnetic field strength demarcation point that ignores the stress signal and finding the optimal magnetic field strength for stress detection are crucial to strong and weak magnetic stress detection.

**Figure 9:**
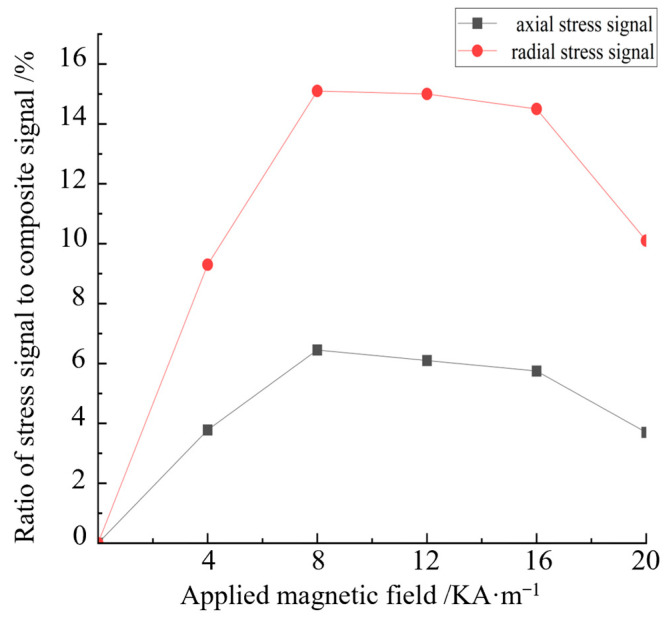
Proportion curve of the radial stress signal.

Under the same applied magnetic field, the extreme value of the composite signal of the stressed steel plate is subtracted from the composite signal extreme value of the stressless steel plate to represent the signal generated by the stress action under the applied magnetic field. The ratio of the stress action signal to the composite signal is the proportion of the stress signal. The proportion of axial and radial component stress signals with the applied magnetic field is calculated separately, as shown in Figure 9.

Figure 9 indicates that when the applied magnetic field is 0–8 KA/m, the proportion of the stress signal increases with the increase in the applied magnetic field strength. When the magnetic field strength is 8 KA/m, the stress signal accounts for the largest proportion: the axial stress signal accounts for 7.5%, and the radial stress signal accounts for 15%. When the magnetic field strength continues to increase to more than 8 KA/m, the proportion of the stress signal gradually decreases. The proportion of the stress signal increases initially then decreases with the increase in the applied magnetic field, which proves the correctness of the theoretical model. The ideal detection magnetic field strength for strong and weak magnetic detection is determined to be 8 KA/m. This result provides a scientific basis and guidance for the study of strong and weak magnetic stress detection.

### 3.4. Calculation of the Stress Magnetic Signal’s Characteristics and the Stress Relationship

The influence of stress magnitude on the characteristic value of the stress magnetic signal is further studied by changing the applied stress magnitude in the environment where the applied magnetic field is unchanged. Simulation calculation is conducted based on the detected ideal magnetic field strength.

**Figure 10:**
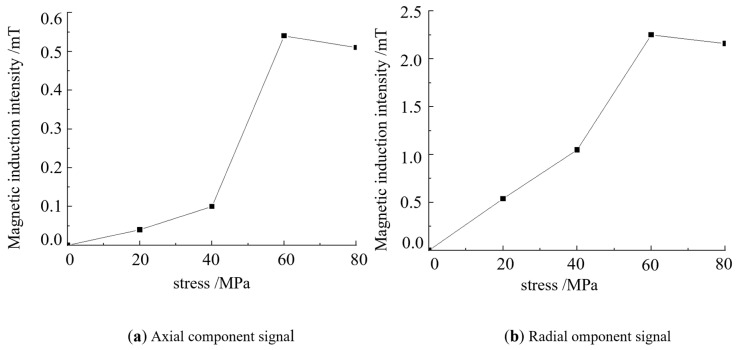
Eigenvalues of composite signals under different stresses.

The simulation is conducted under an applied magnetic field strength of 8 KA/m, and the magnetic induction intensity changes under different applied stresses are examined. Stress values of 0, 20, 40, 60 and 80 MPa are applied to both ends of the Q235 steel defect model, and the applied magnetic field value is used as the ideal detection magnetic field strength to uniformly excite the steel plate. The external uniform magnetic field strength along the length of the steel plate in the air layer is set to 8 KA/m. The minimum value of the axial signal and the maximum value of the radial signal under different stresses are extracted, and the influence of stress on the composite magnetic signal at the defect site is obtained, as shown in Figure 10.

Figure 10a indicates that under the optimal detection magnetic field strength, the axial magnetic signal at the defect increases linearly with the increase in stress. Figure 10b shows that the radial magnetic signal at the defect increases linearly with the increase in stress. In the excitation environment with 8 KA/m stress, with the increase in stress, the magnetic signal feature at the defect increases, that is, with the increase in stress, the magnetic signal feature in the stress-concentration region increases. It is concluded that the stress enhances the magnetic signal feature, and the enhancement trend within a certain range is close to being linear.

## 4. Stress Damage Magnetic Memory Detection Experiment

### 4.1. Experiments

With the pipeline material Q235 steel specimen, a strong and weak magnetic scanning stress detection experiment under the action of pipe stress is designed, and the characteristics of the magnetic induction intensity signal under different stress conditions are experimentally studied. The material of the experimental specimen is Q235 mild steel. The specimen size is 600 mm in length, 60 mm in width, 15 mm in thickness, 60 mm in length at the centre, 2 mm in width at the centre, and 2 mm in depth through the defect.

Strong and weak magnetic stress detection technology is applied for the internal detection of pipelines. The detector is powered by the pipeline medium and scans the pipe wall in the pipeline for defect stress detection. In this study, the steel plate of the pipeline Q235 steel specimen is selected to simulate the actual signal detection state, and grooves are cut out on the surface to simulate the defect. A tensile machine is employed to apply stable tensile stress to both ends of the specimen and simulate the stress concentration of the pipeline. The strong and weak magnetic probe is suspended on the hydraulic lifting device and moved, and the magnetic signal is scanned in the axial direction in the pipeline to determine the position of the stress-concentration zone in the specimen.

The control and adjustment of the applied magnetic field are achieved through a rectification and filtering circuit. By adjusting the rheostat, the excitation current is varied, thereby inducing corresponding changes in the applied magnetic field. In the excitation system, a current of 1 A generates an applied magnetic field of 4 kA/m. The principle of AC demagnetization involves placing the specimen in an alternating magnetic field and using the decreasing hysteresis loop to demagnetize it. The same set of coils is used for both excitation and demagnetization; during the experiment, it is sufficient to switch between the excitation and demagnetization systems via the circuit of the demagnetization system.

**Figure 11:**
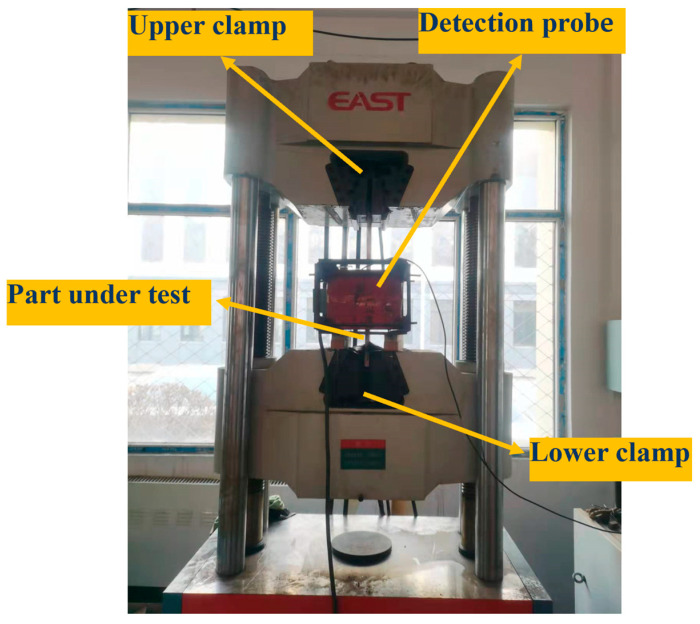
Strong and weak magnetic scanning test platform.

The pipe specimen is fixed on the fixture of the tensile machine, as inaccurate positioning of the specimen may lead to measurement errors. The detector is fixed on the excitation coil to demagnetise the specimen; thorough demagnetization is essential to avoid affecting the accuracy of the detection data. The control hydraulic device drives the detection probe and excitation coil to move at a uniform speed from top to bottom and records the magnetic probe signal acquisition data. When the detection sensor reaches the bottom of the steel plate, it indicates the completion of the initial scanning phase. At this point, the excitation coil is switched to the demagnetization mode. Upon the detection sensor reaching the top of the steel plate, the demagnetization of the entire steel plate is deemed complete, and the detector is returned to its initial position in preparation for the subsequent scan. Figure 11 presents a schematic of the experimental platform for strong and weak magnetic scanning detection.

**Figure 12:**
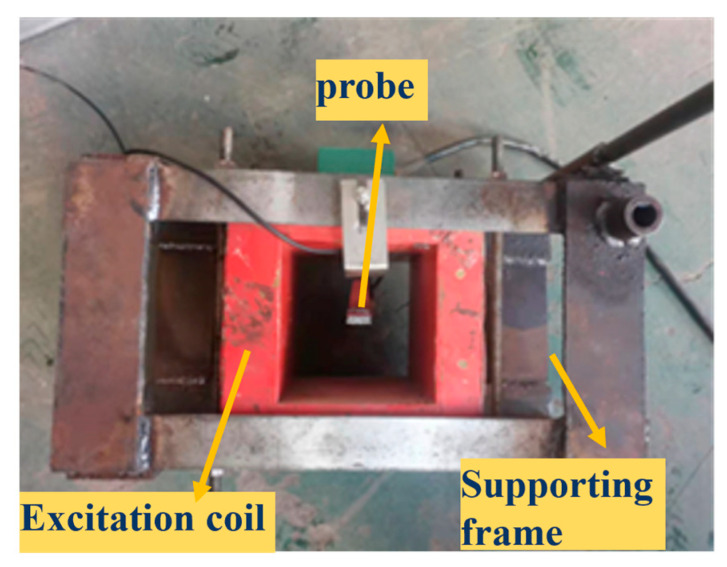
Strong and weak magnetic detector probe.

The strong and weak magnetic signal detection system is shown in Figure 12. It is composed of a strong and weak magnetic signal Hall sensor, a signal storage unit, a host computer system and a hydraulic transmission device, which completes the moving detection mode of the magnetic induction intensity signal of the pipe specimen.

### 4.2. Results and Analysis

Before each repeated scanning detection step, the excitation coil current is increased by 1 A to increase the excitation magnetic field by 4 KA/m each time. The scanning detection step is repeated until the excitation coil current increases to 10 A. A tensile stress of 60 MPa is applied to the steel plate by a tensile machine, and the magnetic signal experimental data with a stress of 60 MPa are obtained after the stress has stabilised.

**Figure 13:**
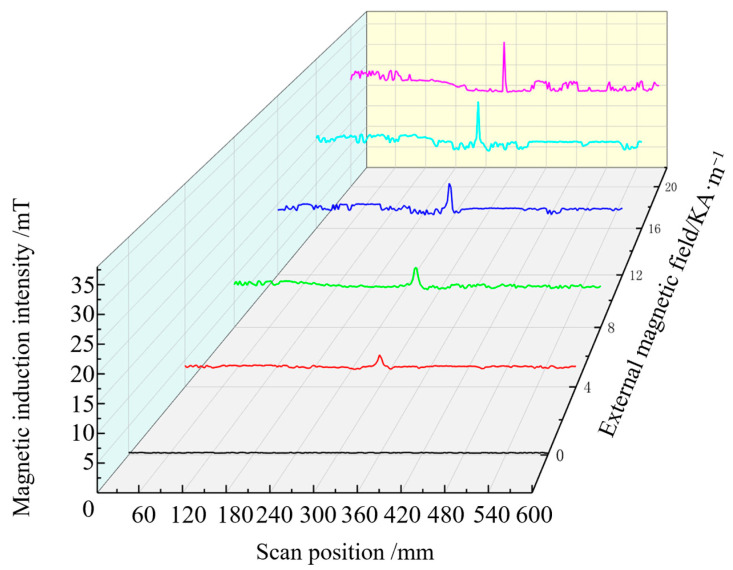
Magnetic signal of the axial component detected by scanning.

**Figure 14:**
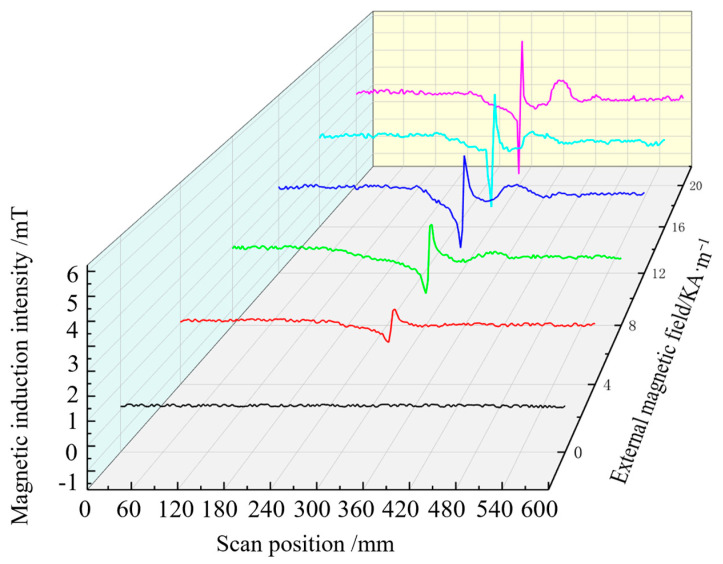
Magnetic signal of the radial component detected by scanning.

Experimental results show that, under a tensile stress of 60 MPa and an applied magnetic field ranging from 0 KA/m to 20 KA/m, the acquired stress composite axial component signal at the defect is presented in Figure 13, while the acquired stress composite axial component signal at the defect under these conditions is shown in Figure 14.

**Figure 15:**
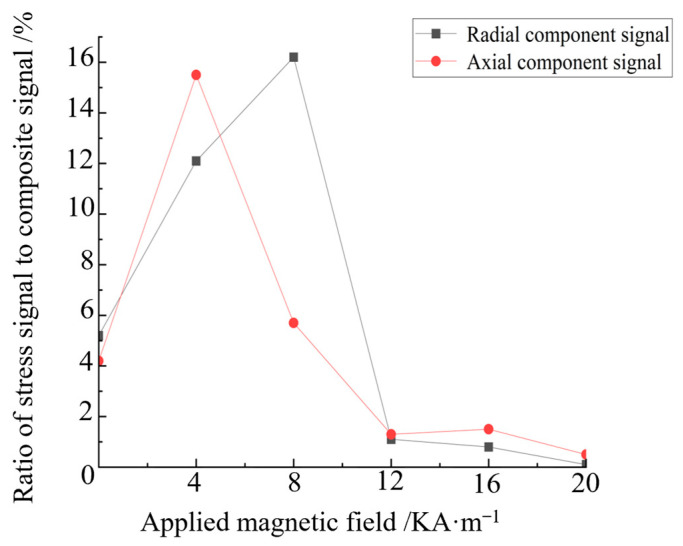
Ratio of the stress signal and variation in the applied magnetic field.

**Figure 16:**
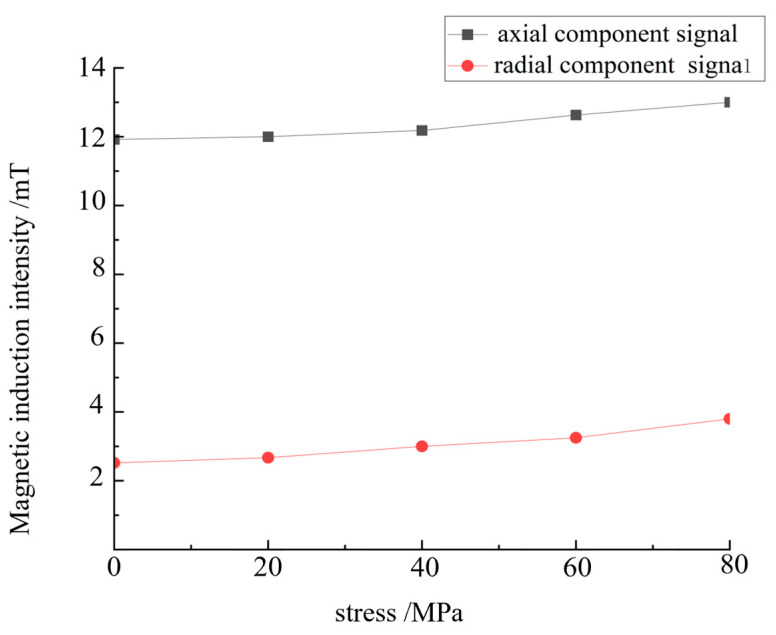
Relationship between the magnetic signal and stress.

Figure 15 and Figure 16 indicate that the axial component signal of the defect and stress composite has a single peak value, and the radial component signal has a peak-to-peak value. As the magnetic field strength increases, the signal strengths of the axial and radial components increase. With the increase in the applied magnetic field, the magnetic signal characteristics can be detected at the pipe defect.

The magnetic induction intensity generated by stress, that is, the stress signal, is obtained by extracting the maximum values of the axial and radial component signals under different applied magnetic field defects with no stress and with 60 MPa stress. The ratio of the stress signal to the composite signal reflects the recognition degree of the stress signal under different applied magnetic fields. The change in the stress signal proportion with the applied magnetic field is calculated, and the result is shown in Figure 15.

As indicated in Figure 15, with the increase in the applied magnetic field, the proportion of the difference in the axial component initially increases then decreases; the maximum value is reached at 4 KA/m. As shown in Figure 15, with the increase in the applied magnetic field, the proportion of the difference in the radial component also increases initially then decreases, and the maximum value is reached at 8 KA/m. The axial signal is consistent with the radial signal. Given that the difference in the magnetic induction intensities of the different stresses is caused by the influence of the different stresses on the pipe specimen, the proportion of the difference can be understood as the stress recognition degree, that is, the recognition degree of the stress signal increases initially then decreases with the increase in the applied magnetic field until the excitation current is adjusted so that the stress signal recognition degree is nearly 0. When the applied magnetic field is 20 KA/m, the maximum point of stress recognition is reached; this magnetic field can be set as the optimal detection field strength in strong and weak magnetic detection, and the value of 8 KA/m coincides with the theory and finite element calculations and has consistency.

When the excitation magnetic field of the strong and weak magnetic scanning detection experiment is set to 8 KA/m, that is, the excitation current is 2 A, the stress signal has the largest proportion. The excitation magnetic field is set to 8 KA/m to study the influence of stress on the magnetic detection signal under the optimal magnetic field. A strong and weak magnetic scanning experiment is performed, and the relationship between the magnetic signal and stress is obtained by analysing the relationship between the maximum value of the radial and axial signals and the applied magnetic field, as shown in Figure 16.

Figure 16 shows that with the strong and weak magnetic field detection method, the change law of the magnetic signal of stress action at the defect can be qualitatively identified. With the increase in the applied load, the composite magnetic signal of the defect and stress and the strength of the identified stress signal are enhanced, which is consistent with the experimental result that stress can increase the magnetic signal. It is also consistent with strong and weak magnetic detection theory and finite element simulation calculation, thus proving that the strong and weak magnetic stress detection method can identify the scientific nature of the stress signal at the defect. The detection results characterise the influence of stress on strong and weak magnetic signals and reflect the strong and weak magnetic effects of stress damage.

## 5. Conclusions

As a new technology, internal stress detection at strong and weak magnetic defects can realise stress identification of pipeline defects and can make up for the shortcomings of traditional in-pipeline detection technology. Utilizing stress detection can yield precise stress distribution data, which aids in a more accurate assessment of material performance. This technique is applicable to a diverse array of materials and structures. Under the action of stress, the internal magnetic domain structure of the ferromagnet changes, and the change in the pinning coefficient is the fundamental reason for the different stress performance of different magnetic fields.

With the optimised J-A model, the relationship between stress and magnetic induction intensity is calculated and analysed, and the law of how magnetic induction intensity increases with the increase in stress is obtained. With the increase in the applied magnetic field, the stress on the magnetic signal initially increases then decreases. The optimal detection magnetic field strength is calculated to be 8 KA/m, and the signal generated by 60 MPa of stress reaches 80 mT. Thus, the relationship between stress and magnetic induction intensity increases linearly.

According to the results of the strong and weak magnetic scanning detection experiment, the axial component signal of magnetic induction intensity has a single peak value, and the radial component signal has a peak-to-peak value when the strong and weak magnetic probe scans the defect position in the pipe specimen. The maximum value of the component signal increases with the increase in the applied magnetic field strength. When the applied magnetic field is 8 KA/m, the difference between the magnetic signals with and without stress is the largest. The experiment on the force–magnetic relationship under the optimal detection of magnetic field strength reveals that the magnetic induction component signal reaches its maximum value at the defect of the pipe specimen. Under the optimal detection of magnetic field strength, the maximum value of the magnetic signal component increases linearly with the increase in the stress of the pipe specimen, and this value can be used as a basis for force–magnetic coupling detection.

## Data Availability

The original contributions presented in the study are included in the article.
